# Case Report: Surgical Correction of a Cystic Duct Stump Leakage Following Cholecystectomy Using an Autologous Rectus Sheath Graft in a Dog

**DOI:** 10.3389/fvets.2021.584975

**Published:** 2021-02-01

**Authors:** Hyun-Jung Han, Kyu-Cahng Kim, Hun-Young Yoon

**Affiliations:** ^1^Department of Veterinary Emergency and Critical Care, Konkuk Veterinary Medical Teaching Hospital, Konkuk University, Seoul, South Korea; ^2^Department of Veterinary Surgery, College of Veterinary Medicine, Konkuk University, Seoul, South Korea

**Keywords:** autologous rectus sheath, bile peritonitis, cholecystectomy, cystic duct stump leakage, graft, yorkshire terrier

## Abstract

A 2.7 kg, 13-year-old, castrated male Yorkshire Terrier was presented with bile peritonitis after cholecystectomy. Exploratory coeliotomy to identify and correct bile leakage revealed that the transected end of the cystic duct was open with no *in-situ* ligatures or vascular clips. The residual cystic duct stump was too short to ligate or seal directly. An autologous rectus sheath graft, harvested from the internal leaf of the rectus sheath, was used to patch the cystic duct stump. The graft was secured over the open duct using several simple interrupted sutures and covered with an omentalization. The clinical signs resolved after surgery, except for a transient increase in hepatobiliary enzyme levels and intrahepatic bile duct dilatation. The enzyme levels returned to near normal on day 25 after surgery. No intrahepatic bile duct dilatation was detected on day 55 after surgery. The owner was contacted for 3 years post-operatively and reported that the dog remained healthy without any long-term complications. Grafting using autologous rectus sheath can be used to treat cystic duct stump leakage that cannot be managed with direct closure using traditional modalities due to spatial constraints.

## Background

Post-operative cystic duct stump leakage (CDSL) resulting in bile peritonitis is a reported complication of open and laparoscopic cholecystectomy in humans and animals (1–4). CDSL accounts for 3–8.7% of complications associated with cholecystectomy in dogs (2, 5). Most CDSL in veterinary medicine is iatrogenic and due to technical failures, such as dislodgement of an improperly sized or improperly placed surgical clip (6, 7). For surgical treatment of CDSL in dogs, direct closure of the remaining cystic duct by ligation using suture or clips and a vessel sealing device is the most common way to close the CDSL (2, 6, 7). In cases where direct closure is not possible or poses a high risk of poor outcome, alternative approaches are needed.

This case report describes the surgical technique and outcome of the treatment of CDSL following open cholecystectomy in a Yorkshire Terrier. An autologous rectus sheath (ARS) graft was used to completely seal the CDSL, which could not be managed with direct ligation because of spatial constraints.

## Case Presentation

A 2.7 kg, 13-year-old, castrated male Yorkshire Terrier dog was referred for further evaluation and treatment of suspected bile peritonitis following open cholecystectomy. According to the referring veterinarian, the dog had been diagnosed with gallbladder mucocele, which had not been ruptured, and underwent open cholecystectomy with single circumferential ligation of the cystic duct using 3–0 polyglyconate (Maxon, Covidien, Mansfield, Massachusetts) 2 days before referral. The CBD lavage was performed by antegrade flushing without duodenotomy. The veterinarian also reported that abdominal fluid aspirated 2 days after the open cholecystectomy, exhibited a total bilirubin (TBIL) level of 27.9 mg/dL, which was at least 20 times the level in the serum (1.1 mg/dL; reference range, 0–0.4 mg/dL). At this time, the dog was referred to the department of veterinary emergency and critical care in the veterinary medical teaching hospital.

Physical examination on presentation, revealed anorexia, depressed mentation, abdominal distension with pain on palpation, normothermia, mild dehydration, and yellow skin. A white blood cell count revealed mild leukocytosis (WBC, 20.94 × 10^3^/μL; reference range, 6–17 × 10^3^/μL), with about 90% mature neutrophils but no band neutrophils. Serum biochemical abnormalities included mild hypokalemia (3.6 mmol/L; reference rage, 3.8–5.0 mmol/L); high concentrations of alanine transaminase (ALT, 205 U/L; reference range, 19–70 U/L), aspartate transaminase (AST, 140 U/L; reference range, 15–43 U/L), alkaline phosphatase (ALKP, 510 U/L; reference range, 15–127 U/L), lactate dehydrogenase (174 U/L; reference range, 0–130 U/L), creatine kinase (1512 U/L; reference range, 46–320 U/L), gamma-glutamyltransferase (GGT, 9.6 mg/dL; reference range, 0–6 mg/dL), TBIL (8.97 mg/dL; reference range, 0–0.4 mg/dL), and C-reactive protein (CRP, > 210 μmol/L; reference range, <35 μmol/L); and low concentrations of creatinine (0.2 mg/dL; reference range, 0.5–1.3 mg/dL) and total protein (4.6 g/dL; reference range, 5.4–7.4 g/dL). Coagulation profiles showed prolonged activated partial thromboplastin time (19.2 s; reference range, 14–18 s) and mildly elevated D-dimer concentration (0.4 μg/mL; reference range, <0.3 μg/mL). Preoperative diagnostic imaging including abdominal radiography and ultrasonography confirmed ascites. Biochemical analysis of the abdominal fluid revealed a markedly high TBIL level (74.33 mg/dL) relative to that in the serum (8.97 mg/dL). The abdominal ultrasonography showed no obstruction or distention of the CBD and hepatic duct. On the basis of those findings, the patient was diagnosed with bile peritonitis due to post-operative bile leakage.

Coeliotomy was performed to identify and repair the post-operative bile leakage. The patient was premedicated with enrofloxacin (5 mg/kg subcutaneously), butorphanol [0.2 mg/kg intravenously (IV)], and midazolam (0.1 mg/kg IV). General anesthesia was induced with propofol (4 mg/kg IV) and maintained with isoflurane in oxygen after endotracheal intubation. After aseptic preparation, a ventral midline coeliotomy was performed in a routine fashion.

**Figure 1:**
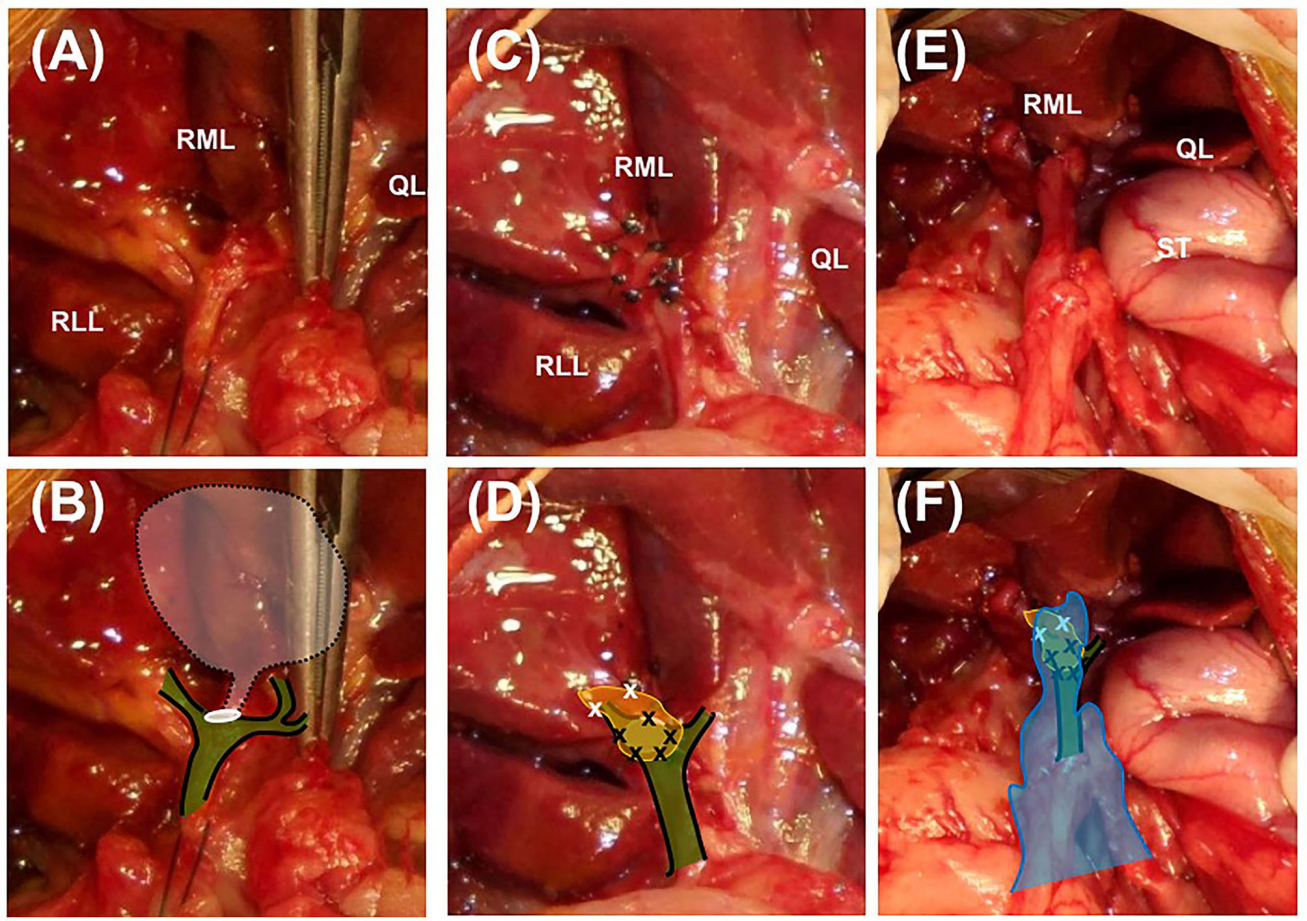
Closure of the cystic duct stump using an autologous rectus sheath graft in a Yorkshire Terrier. Images **(A,C,E)** are intaoperative photographs; **(B,D,F)** incorporate schematic overlays to highlight the relevant anatomy and location of suture placement. **(A,B)** The transected end of the cystic duct is completely open (white circle) and is close to the base of the bilateral hepatic ducts and the common bile duct (green color plane with black solid line). The white semi-transparent plane with black dotted line indicates the location of the removed gall bladder and cystic duct. **(C,D)** The 1 × 1 cm rectus sheath graft (yellow plane) is sutured to the end of the cystic duct stump with simple interrupted sutures using 5–0 polyglyconate (black × marks). The abundant dorsal part of the sheath is sutured to the fibrous capsule of the surrounding right medial liver lobe (white × marks). **(E,F)** After confirmation of complete closure of the cystic duct, greater omentum is tacked over the patched cystic duct stump by suturing with the fibrous capsule of the right medial liver lobe (blue plane). RML, right medial lobe; RLL, left lateral lobe; QL, quadrate lobe; ST, stomach.

**Figure 2:**
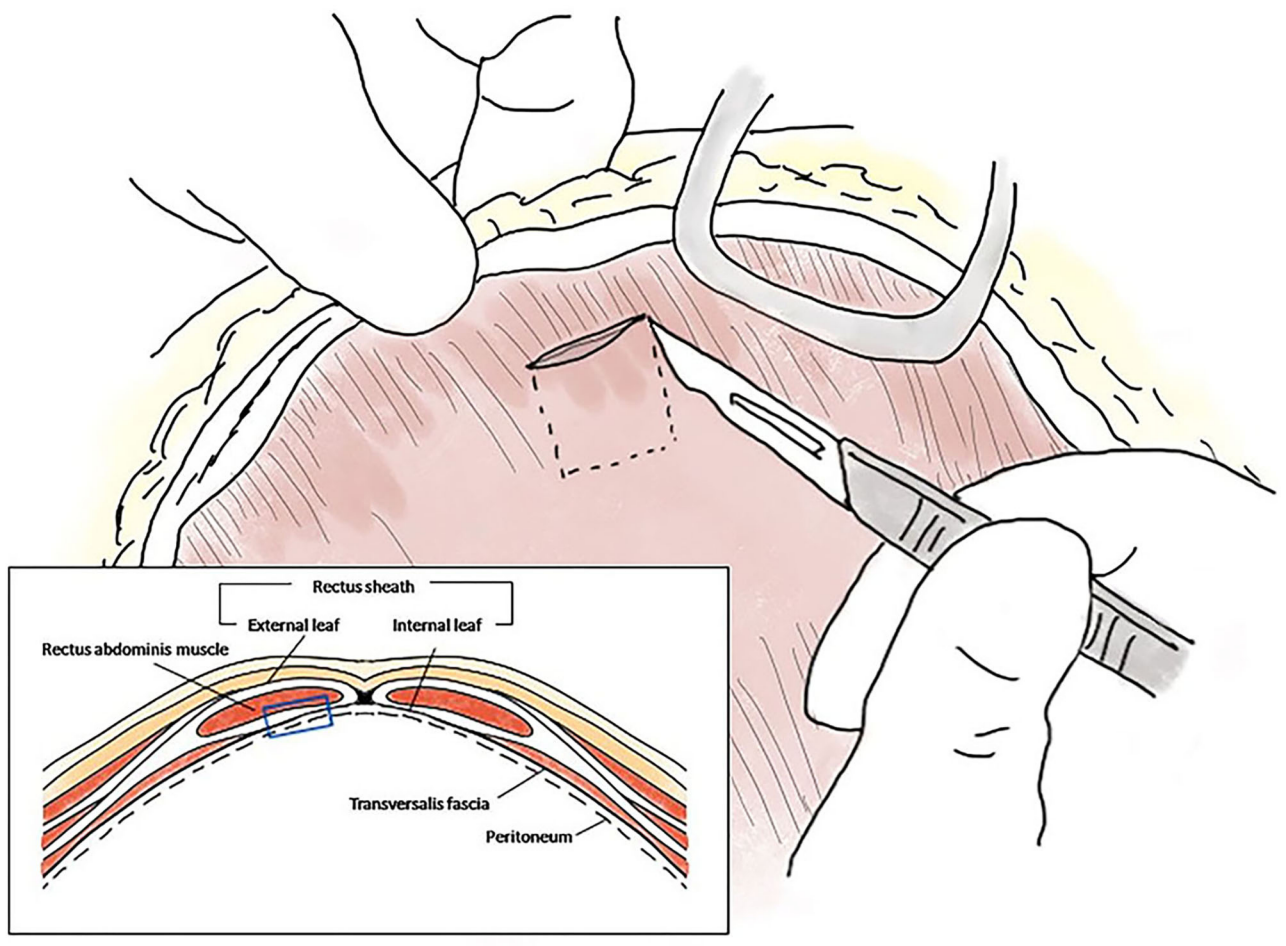
Schematic diagram of the autologous rectus sheath graft. The internal leaf of the rectus sheath was approached via the coeliotomy site, and a 1 × 1 cm sheath with peritoneum was peeled off at the level of the midline, ~1 cm from the incision (blue empty square in the box).

A moderate amount of diffuse, bile-stained effusion was identified throughout the abdomen. After the abdominal effusion was sampled for bacterial culture, the effusion was removed by constant suction. After careful approach to the gall bladder fossa and CBD, complete transection of the cystic duct was identified at the junction of the cystic duct and the CBD (Figures 1A,B). The end of the cystic duct was completely open without any ligatures. A tied suture had slipped from the transected cystic duct and was found separately. Other visible hepatic ducts and the CBD were intact without bile leakage. CBD patency was confirmed by antegrade cannulation, from the transected end of the cystic duct through the common bile duct into the duodenal papilla, and saline flushing with a 5 Fr feeding tube [F.D.T. (D), HMS Inc]. There was no remnant of the cystic duct stump (CDS) to be ligated, because the almost entirely cystic duct was transected, and the severed end was very close to the bilateral hepatic ducts and the CBD. The length of CDS remnant seemed to be <1 mm that could not be even measured accurately. Direct closure of the CDS could not be achieved with any traditional method using cystic duct ligatures, oversewing sutures, surgical clips, or vessel sealing devices without damaging the CBD. Therefore, the open end of the CDS was covered with an autologous graft. For the autologous free graft, 1 × 1 cm of the internal leaf of the ARS was harvested at the middle of the abdomen (Figure 2). The graft was capped over the CDS and attached to the CDS with several simple interrupted sutures using 5–0 polyglyconate (Maxon, Covidien, Mansfield, Massachusetts), facing the peritoneum to the lumen (Figures 1C,D). The sutures were placed outside the lumen, with care taken to impinge the hepatic ducts as little as possible. After the graft was sutured to the CDS, an abundant portion of the dorsal part of the graft remained outside the suture in the gallbladder fossa and was additionally sutured to the serous and fibrous capsule over the right medial liver lobe surrounding the CDS. After confirming that there was no bile leakage from the grafting site over a period of 5 min, omental graft was performed by suturing the omentum to the fibrous capsule of the liver lobe and other soft tissue surrounding the CDS with several simple interrupted sutures using 5–0 polyglyconate (Maxon, Covidien, Mansfield, Massachusetts) (Figures 1E,F). The abdominal cavity was then copiously lavaged with warm saline and closed routinely.

**Figure 3:**
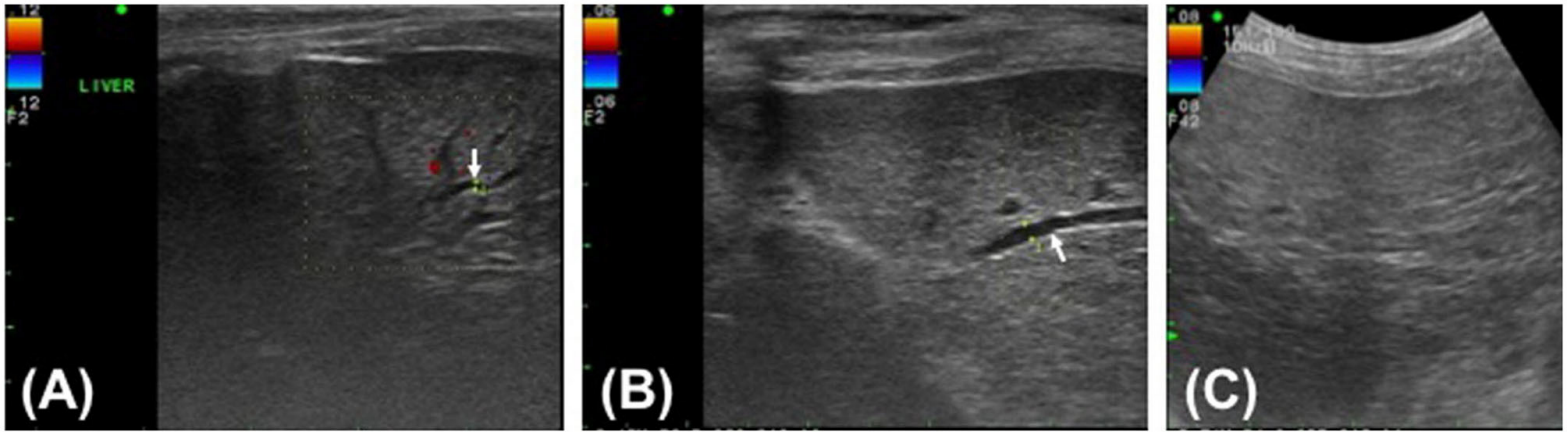
Post-operative ultrasound images of the hepatic parenchyma of a Yorkshire Terrier with unusual cystic duct stump leakage treated using an autologous rectus sheath graft. Color Doppler imaging shows intrahepatic bile duct dilatation in images **(A,B)** (white arrow). The intrahepatic bile duct diameter is 0.7 mm on post-operative day 5 **(A)**, 1.4 mm on post-operative day 25 **(B)**, and not measurable on post-operative day 55 **(C)**.

After recovering uneventfully from anesthesia, the dog was quiet, alert, and responsive while maintaining normal vital signs and normotension. The dog ate prescription canned diet (i/d) spontaneously starting on the day after surgery. Postoperative medication included continuous infusion of fentanyl (4 μg/kg/h) and lidocaine (50 μg/kg/h) for the first 24 h after surgery, followed by carprofen (2.2 mg/kg orally twice daily) and tramadol (5 mg/kg orally twice daily) for 5 days, maropitant (0.1 mL/kg subcutaneously once daily) for 4 days, and pantoprazole (1 mg/kg IV once daily), enrofloxacin (5 mg/kg IV twice daily), and metronidazole (15 mg/kg IV twice daily) for 7 days. The systemic antibiotics were administered pending culture results and were discontinued when the culture of the abdominal fluid revealed no bacterial growth. As hepatic supplements, ursodeoxycholic acid (10 mg/kg orally once daily) and s-adenosylmethionine/silybin (zentonil 20 mg/kg orally once daily) were administered for several months after surgery. Postoperative abdominal ultrasound showed intrahepatic bile duct dilatation with a diameter of 0.7 mm on day 5 after surgery and 1.4 mm on day 25 after surgery (Figures 3A,B). No dilatation was detected by ultrasound on day 55 after surgery (Figure 3C). Ultrasound revealed minimal residual abdominal effusion immediately after surgery, which subsequently disappeared. All serum liver enzyme levels were increased immediately after surgery compared with the levels prior to surgery, except for the TBIL level, which decreased post-operatively but was still higher than the normal range (ALT, 311 U/L; AST, 316 U/L; ALKP, 781 U/L; TBIL, 2.99 mg/dL). All levels had returned to near normal levels on day 25 after surgery (ALT, 117 U/L; AST, 24 U/L; ALKP, 309 U/L; TBIL, 0.12 mg/dL). The GGT level exhibited a transient post-operative increase (15.1 mg/dL) and subsequently fell to near the normal range by 3 days after surgery (8.6 mg/dL). The GGT level was again increased to 73.5 mg/dL on day 25 after surgery but had returned to normal level on day 55 after surgery (5.2 mg/dL). Inflammatory markers, including WBCs and CRP, consistently improved after surgery and had returned to within reference ranges on day 25 after surgery (WBC, 11.42 × 10^3^/μL; CRP, 38 μmol/L).

For 6 months after surgery, the dog's general condition and hepatobiliary enzyme levels were re-examined monthly or bimonthly at the Konkuk University Veterinary Medical Teaching Hospital. No significant findings were noted during that period. Telephone follow-up was conducted for 3 years following the surgery. The owner reported that the dog remained healthy during that period without evidence of clinical signs associated with hepatobiliary disease.

## Discussion

Because untreated CDSL can induce fatal complications associated with bile leaks in humans and animals, such as bile peritonitis and hepatic inflammation (2, 4, 8), immediate detection and repair of CDSL is a key factor in positive outcomes (8). Some differences in the management of CDSL between humans and dogs have been reported. In humans, despite significant morbidity and mortality caused by bile peritonitis, early detection and minimally invasive treatment of CDSL can result in a good prognosis (9). Diversion of the biliary flow by endoscopic methods, including sphincterotomy with or without stenting and nasobiliary drain placement for reduction of bile flow resistance, are the preferred treatments for CDSL in humans (4, 10). Those treatments facilitate spontaneous healing of the cystic duct, resulting in a 94–100% cure rate (3, 4, 9). Re-exploratory surgery has an 83–100% success rate in human patients, but it is not commonly selected because of its invasiveness (4). In dogs, the cure rate and mortality of patients with CDSL have not been investigated, but the mortality rate is 28–39% among dogs that are treated surgically for bile peritonitis (5, 11). For the treatment of CDSL in dogs, biliary diversion surgery has not been well-developed because of technical challenges and the unclear relationship between increased CBD pressure and CDSL (12). Therefore, open surgical treatment remains the preferred approach for CDSL in dogs (2, 6, 13).

For open surgical treatment of CDSL, direct closure using suture, surgical clips, or a vessel sealing device has been introduced into the veterinary field to seal the cystic duct (2, 6, 7). If there is a spatial constraint in the CDS, however, treatment by direct closure can have a high risk of slippage in case of suture ligation. Furthermore, if the cystic duct is ligated, clipped, or sealed too distally, the proximal CBD can be compromised, resulting in obstruction of the hepatic duct tributaries (4, 14). In this case report, the CDS was too short to be ligated or sealed, and the hepatic ducts were located very close to the transected end of the cystic duct and therefore highly likely to be damaged by direct closure of the CDS. A previous study reported that it is feasible to sacrifice one or more hepatic ducts without removing the affected liver lobe because collateral drainage will develop (2). In this case report, the two most proximal bilateral hepatic ducts were involved, however, accounting for more than 50% of the total hepatic ducts in the animal. Because dogs usually have a total of 3 or 4 major hepatic tributaries (15), impingement or stricture of the hepatic ducts might have made the overall hepatic flow insufficient, resulting in clinically significant cholestasis. Because of those risks, direct closure was not performed. As a result, there was no possibility of ligation slippage, and cholestasis caused by hepatic duct stricture was avoided, except for transient cholestasis due to post-operative biliary edema.

Bile flow diversion, as used in humans, was not considered because of the small size of the dog and the luminal patency of the CDS. No biliary stent or tube for diversion small enough to be applicable to the dog was available, and even if bile flow diversion was possible, it was not considered the best option, because diversion would not completely prevent bile leakage from the open cystic duct.

As an alternative option for CDSL closure, an autologous graft was used to cover the transected end of the cystic duct. That grafting for CDSL treatment is the most inventive part of this case report, because there are no other reports of such grafting attempted in a dog with CDSL, although there have been reports of the use of biomaterials or grafts to repair CBD injury by proximal and distal end-to-end anastomosis (16–24) or primary closure of choledochal wall defects (25, 26). In order to attach the autologous graft to the CDS, the graft was sutured with simple interrupted 5–0 absorbable monofilament, similarly to previous reports in which interrupted 5–0 to 6–0 absorbable or non-absorbable suture was used to patch the graft to the CBD (16, 17, 19, 22). The use of sealant to prevent leakage from the grafting site was not considered, because a previous study reported that a fibrin sealant could act as an infectious nidus, causing post-grafting acute cholangitis (17). ARS grafting to the circumference of the open end of the CDS, patching of the remnant of the graft to the surrounding fibrous capsule, and traditional omental grafting were enough to seal the short CDS completely without any bile leakage form the grafting site.

No implant to prevent post-grafting CBD stricture was placed, because there was a low possibility of post-grafting stricture, considering the anatomical location of the graft. According to previous studies, a graft that bridges a CBD defect is likely to develop post-grafting CBD stricture, so implants such as a biliary stent or tube are usually recommended to prevent stricture (16–22, 24, 26, 27). On the other hand, because the graft in this case report was placed in the CDS, which was at the proximal end of the CBD and not the middle of the CBD, post-grafting CBD stricture was not a major concern, because a graft at the proximal end of the CBD is unlikely to impede the bile flow through the CBD even if stricture occurs.

ARS graft for the reconstruction of the bile duct in humans was first reported in 1917 (23). Several experimental applications of ARS graft in dogs were reported between 1998 and 2005 (16, 24, 26). ARS graft was highly successful with a 83–91% success rate in experimental dogs in several previous studies (16, 24, 26). Those clinical and experimental trials resulted in reconstructions of the biliary tract with no leakage, grossly similar appearance between the grafts and the bile duct, and biliary columnar epithelium covering the inner surface of the grafts (16, 23, 24, 26). The same morphological change of ARS graft tissue was observed in a study that applied ARS graft for arterial replacement in a dog model (28). In that study, the fibroblast cells of the ARS graft changed into myofibroblast cells similar to the smooth muscle cells of intact arteries. Thus, several previous studies demonstrated morphological and functional remodeling of ARS grafts, which became similar to the host vessels via arterialization (28). Fascial grafts have other benefits as well, including ease of harvesting, low rates of adverse reaction, and lower cost compared with synthetic grafts, which are more thrombogenic, more susceptible to infection, and more likely to be rejected than fascial grafts (20). In this case study, the ARS graft was readily available and relatively easily harvested in a situation that arose quickly and unexpectedly. On the basis of the results from previous studies, the peritoneum attaching to the internal leaf of the ARS was expected to promote biliary epithelization of the graft, helping to settle the graft to the CDS. Unfortunately, it was not possible to histologically confirm the ARS remodeling because of the invasive procedure that would be required in order to do so.

There were no major post-operative complications, except for post-operative cholestasis due to the increased levels of ALT, AST, ALKP, and GGT and the dilatation of the intrahepatic bile ducts after surgery. The TBIL level rapidly decreased after the surgery but was still higher than the normal range. Post-grafting cholestasis can be caused by post-operative biliary edema, post-operative CBD stricture, or implant-associated problems such as biliary stent migration and T-tube obstruction due to bile sludge and stones (17, 19). Because there was not any implant inside the patient's biliary tract, and the TBIL level and size of the intrahepatic bile ducts returned to normal after the surgery, implant-associated problems and post-operative stricture could be ruled out. Therefore, post-operative biliary edema was considered to be the most likely cause of the temporary cholestasis after surgery. A previous study suggested that post-operative biliary edema could be caused by perioperative biliary manipulation and suture placement, finally leading to stenosis and elevation of the hepatobiliary enzyme levels. The stenosis and elevated enzyme levels were usually temporary, however, with enzymes returning to normal levels within 3 weeks (17). In this case report, considerable manipulation of the CBD was unavoidable during surgery, so there was a strong possibility of post-operative biliary edema leading to transient stenosis of the biliary tract. Because it was a transient event, dilated intrahepatic bile ducts were no longer observed on the ultrasound performed on day 55 after surgery. Furthermore, most of the hepatobiliary enzyme levels returned to normal by 25 days after surgery. The transient increase in GGT observed 25 days after surgery might have been associated with biliary tract hyperplasia or waxing and waning of cholestasis. It was not considered a major complication, however, because there were no associated clinical signs or abnormal findings consistent with increased GGT. Therefore, post-operative cholestasis should be considered an inevitable, short-term, non-life-threatening complication of the surgery.

Antibiotics were used preoperatively and post-operatively because of the possibility of infectious peritonitis from bile leakage. There was concern that the patient might have resistance to penicillin and cephalosporin antibiotics, because the patient had a history of long-term administration of clavamox and first-generation cephalosporins to manage dermatitis and otitis externa. Therefore, fluoroquinolone was selected as an alternative drug to cover Gram-negative bacilli and Gram-positive cocci. Fluoroquinolone is one of the most commonly used drugs for bacterial cholangitis, and resistance to fluoroquinolone in *Escherichia coli* is less common than resistance to penicillin and cephalosporins, although there is a risk that fluoroquinolone resistance will develop over time (29). Because of that risk, fluoroquinolone was used only for a short period, until the post-operative culture results showed that there was no infection.

## Concluding Remarks

This is the first report of the successful treatment of CDSL using an ARS graft in a dog. The grafting technique using an ARS created a seal over the CDS, which could not be directly sealed because of a short residual stump.

## Data Availability Statement

The original contributions presented in the study are included in the article/supplementary material, further inquiries can be directed to the corresponding author/s.

## Ethics Statement

Ethical review and approval was not required for the animal study because this is a case report. Written informed consent was obtained from the owners for the participation of their animals in this study.

## Author Contributions

H-JH contributed to preparing the conception and design of the work, analyzing the data, and writing the manuscript. K-CK assisted with surgery, was involved with aftercare and acquisition of data for the work. H-YY performed surgery and supervised aftercare and critically revised the manuscript. All those designated as authors contributed substantially to the study design, preparation and final approval of the manuscript.

## Conflict of Interest

The authors declare that the research was conducted in the absence of any commercial or financial relationships that could be construed as a potential conflict of interest.
